# Simultaneous Optimization of Multiple Response Variables for the Gelatin-chitosan Microcapsules Containing Angelica Essential Oil

**Published:** 2017

**Authors:** Qiang Li, Li-Jian Sun, Xian-Feng Gong, Yang Wang, Xue-Ling Zhao

**Affiliations:** a *Key Laboratory of Chemical Engineering Process & Technology for High-efficiency Conversion, College of Heilongjiang Province, Harbin 150080, PR China.*; b *School of Chemistry and Materials Science, Heilongjiang University, Harbin 150080, PR China.*

**Keywords:** Microcapsules, angelica essential oil, multiresponse optimization, gelatin, sustained release, antioxidation rate

## Abstract

Angelica essential oil (AO), a major pharmacologically active component of *Angelica sinensis *(Oliv.) Diels, possesses hemogenesis, analgesic activities, and sedative effect. The application of AO in pharmaceutical systems had been limited because of its low oxidative stability. The AO-loaded gelatin-chitosan microcapsules with prevention from oxidation were developed and optimized using response surface methodology. The effects of formulation variables (pH at complex coacervation, gelatin concentration, and core/wall ratio) on multiple response variables (yield, encapsulation efficiency, antioxidation rate, percent of drug released in 1 h, and time to 85% drug release) were systemically investigated. A desirability function that combined these five response variables was constructed. All response variables investigated were found to be highly dependent on the formulation variables, with strong interactions observed between the formulation variables. It was found that optimum overall desirability of AO microcapsules could be obtained at pH 6.20, gelatin concentration 25.00%, and core/wall ratio 40.40%. The experimental values of the response variables highly agreed with the predicted values. The antioxidation rate of optimum formulation was approximately 8 times higher than that of AO. The *in-vitro* drug release from microcapsules was followed Higuchi model with super case-II transport mechanism.

## Introduction

Angelica essential oil (AO), a major pharmacologically active component of *Angelica sinensis *(Oliv.) Diels, possesses hemogenesis, analgesic activities and sedative effect, and finds application in the treatment of a range of conditions including menstrual disturbance and anemia (1). Over 40 compounds have been identified in AO (2) with the major constituent being ligustilide (3). Numerous studies have shown that ligustilide has a neuroprotective effect against ischaemia-reperfusion injury via anti-apoptosis and anti-oxidation in neurons (4-6).

However, the application of AO in pharmaceutical systems has been limited because of its low oxidative stability (7). When AO is processed, used, and stored as a drug or dietary supplement, accelerated degradation of AO can result in decreased drug effect and off-flavor development (8). In addition, the above benefits can be effectively acquired when AO is taken repeatedly and continuously for a given period of time, because of the short retention time of AO in plasma. Thus, the sustained delivery of AO and prevention of AO from oxidation were two challenges facing the oral delivery systems design works. 

Microencapsulation technology was considered as a promising technology to protect from oxidation and maintain sustained release for AO. Complex coacervation, caused by electrostatic attraction of two oppositely charged colloids, has been used widely to microencapsulate drug components (9, 10). The coating materials can be prepared from gums, proteins, polysaccharides, lipids and synthetic polymers (11, 12). However, for a medicinal product, a bio-polymeric system based on natural polysaccharides or protein could provide a safe, inert and effective oral delivery matrix (13, 14). Gelatin B has excellent emulsibility, high hydrophilicity, and particular gelling property (15). Chitosan has been reported to be very suitable for preparation of microcapsules for controlled drug release. The pharmaceutical and food applications of chitosan have remarkably increased over recent years because of its low production costs, biodegradability, biocompatibility and recent FDA approval (16). In this work, the coacervate of gelatin and chitosan was selected as the coating materials. 

A problem facing the product development community is the selection of a set of conditions which will result in a product with a desirable combination of properties. Response surface methodology (RSM) is useful in improvement and optimization of pharmaceutical formulations by finding the analytical relationship between input and output variables considered in experiments. RSM also has the ability to produce an approximate function using a smaller amount of data and fewer numbers of experiments. However, most previous applications based on RSM have only dealt with a single-response problem (17, 18) and multi-response problems have received only limited attention. 

During optimization of gelatin-chitosan microcapsules (GCM), usually several response variables were to be optimized. In many cases, these responses were competing, i.e., improving one response might have an opposite effect on another one, which further complicated the situation. A method to resolving the problem involving the simultaneous optimization of multiple response variables is through the use of a desirability function that combined all the responses into one measurement (19, 20). Studies have showed that a desirability function method is useful in simultaneously optimizing several response variables (21, 22).

In this paper, the effect of formulation variables of GCM containing AO on several response variables were investigated, and subsequently, a formulation with excellent antioxidation and sustained-release properties was obtained using RSM after constructing a desirability function that combined all response variables.

## Materials and Methods

*Materials*

AO was obtained by supercritical carbon dioxide extraction method and donated by Humei Natual Spices Oil Refineries Factory. The natural proportion of ligustilide in AO was 52%. Gelatin from porcine skin, type B, Bloom 260, was obtained from Xiamen Huaxuan Gelatin Co., Ltd. Chitosan, low viscosity (22 cps), 200 thousand Daltons molecular weight, 88.1% deacetylation, was purchased from Shandong AK Biotech Ltd. Glutaraldehyde (50%), as a crosslinker, was provided by Kermel Co., Ltd. The other chemicals and solvents used in this work were of analytical grade, purchased from Sigma-Aldrich Co., Ltd. Deionised water (electrical conductivity < 2 μS cm^-1^) was used throughout all the experiments.

*Preparation of GCM*

GCM were prepared using the complex coacervation method. Gelatin solution (concentrations: 6.34-23.66%, w/w) was prepared by swelling gelatin in deionised water followed by heating (50 ºC) until the appearance of a clear solution. Chitosan was dissolved in 1% (w/v) acetic acid solution by stirring overnight until a clear solution was obtained. The concentration ratio of gelatin to chitosan was 10/1 described by Silva *et al*. (23). O/W emulsion was prepared by adding AO (core/wall ratios: 19.02-70.98%) into the above gelatin solution at high shear (Fluko Homogenizers, model FA25, USA) rate of 10000 rpm (30-40 s), and diluted 2 times with deionised water. Then chitosan solution was added to the emulsion under stirring rate of 400 rpm for 30 min. Initially, the coacervation of chitosan and gelatin was brought about by gradual addition of 2% sodium hydroxide solution. Then the pH of mixture was adjusted to 5.32-6.18. In this stage, GCM were formed. This mixture was cooled to 20 ºC in water-bath for obtaining coacervate precipitation. The cross-linking of the GCM was achieved by addition of a certain amount of 5% glutaraldehyde solution (0.3-0.5 g glutaraldehyde / 1 g wall materials). After 20 min, the mixture was heated and maintained at 30-40 ºC, and stirred for 40 min. This mixture was then cooled to room temperature. The microcapsules were filtered by vacuum pump, then washed with deionised water at 35 ºC to remove excess glutaraldehyde, and then dried.

*Determination of yield*

Yield (%, w/w) was calculated as follows: Yield (%, w/w) = *W*_d_/*W*_s_×100 (eq, 1), where *W*_d_ is the weight of dried microcapsules recovered, *W*_s_ is the total weight of the wall materials and AO initially added during the batch preparation.

*AO assay in microcapsules*

In AO, the relative amount of ligustilide was constant. Therefore, the content of AO in the microcapsules was calculated according to the following formula: *C*_A_ = *C*_L_/*P*_L_×100 (eq, 2), where *C*_A_ is the content of AO, *C*_L_ is the content of ligustilide, *P*_L_ is the natural proportion of ligustilide in AO (i.e., 52%). High performance liquid chromatography (HPLC) method was established for the determination of ligustilide. A series of known concentrations in the range 1.25-100 μg mL^-1^ of ligustilide in mobile phase containing 75% acetonitrile and 25% deionised water were determined at the detective wavelength 326 nm (Shimadzu, model LC-10AT, Japan). A C_18_ column (250 mm × 4.6 mm, 5 μM, Dikma Technologies, China) was used at room temperature, and flow rate was 0.80 mL min^-1^. The respective peak areas were recorded and plotted.

A certain amount of GCM was accurately weighed and dispersed in a known volume of mobile phase. After staying overnight, this dispersion was filtrated, and the content of ligustilide in the resultant filtrate was measured by HPLC method as described above. Based on the resulting value, the content of AO in the microcapsules was calculated. Each experiment was carried out in triplicate.

*Measurement of encapsulation efficiency (EE)*

The EE of the microcapsules was determined as follows: EE (%) = *C*_a_/*C*_i_×100 (eq, 3), where *C*_a_ is the actual drug content in the microcapsules, *C*_i_ is the content of the drug initially added during the batch preparation. 

*Stability of microcapsules against oxidation*

Oxidation is the major degradation pathway of AO (7). The stability of microcapsules against oxidation was studied in a 9-day acceleration test (60 ºC, 75% relative humidity, exposed to air)(24, 25). The samples were collected at regular intervals. AO assay in GCM was determinated at the different time points. For unencapsulated AO, the contents of ligustilide at the various intervals were determined by HPLC as described in “AO assay in microcapsules”, and the contents of unoxidated AO were calculated. Antioxidation rate (AR) was determined by substituting the resulting values in the following expression: AR (%) = *C*_n_/*C*_0_×100 (eq, 4), where *C*_n_ and *C*_0_ are the unoxidated AO content in microcapsules or in unencapsulated AO at Day *n* and Day 0, respectively.

*Release of AO from GCM in-vitro*

The release of AO from the GCM was investigated using a dissolution tester (Huanghai, model ZRS-8G, China) under a constant oar speed of 50 rpm at 37 ºC. The phosphate buffer at pH 7.4 was used as the dissolution media (1000 mL). At appropriate time intervals, 5 mL samples were withdrawn and filtered through a 0.45 μM Millipore membrane filter. The ligustilide concentration in the dissolution media was determined by HPLC as described in “AO assay in microcapsules”, and the concentration of AO was calculated. For unencapsulated AO, the percent of released drug was calculated based on the added amount of AO. For GCM, the percent was obtained based on the results of AO assay in GCM, Then, the percent of released drug was plotted *vs.* time. The percent of drug released in 1 h (P_1_) and time to 85% drug release (t_85_) were calculated.

To evaluate the kinetics of drug release from the microcapsules, *in-vitro *release data were analyzed according to zero-order (eq, 5), first-order (eq, 6), Higuchi (eq, 7), and Korsmeyer-Peppas (eq, 8) equations:

*M*_t_ */M*_∞_=100 (1-*k*_0_*t*)                     (eq. 5)

*M*_t_ */M*_∞_=100 (1-exp(*k*_1_*t*))                      (eq. 6)

*M*_t_ */M*_∞_=*k*_h_*t*^0.5^                      (eq. 7)

*M*_t_ */M*_∞_=*kt*^n^                     (eq. 8)

where *M*_t_*/M*_∞_ is the fractional release of the drug in time *t*, *n* is the release exponent, indicative of the transport mechanism, *k*_0_, *k*_1_, *k*_h_ and *k* are constants incorporating geometrical and structural characteristics of the macromolecular network system and the drug (26, 27). The release exponent for polymeric controlled delivery systems of spherical geometry has values of *n *≤ 0.43 for Fickian release (diffusion-controlled release), 0.43 < *n *≤ 0.85 for non-Fickian release (anomalous transport) and *n *> 0.85 for super case-II transport (relaxation-controlled release) (28). All data were analyzed using OriginPro 8 (OriginLab, UK). 

*Scanning electron microscopy (SEM) of GCM*

The outer structures of the microcapsules were studied by SEM. Dried microcapsules were mounted on metal stubs and coated with gold (20 nm thickness) using an ion coater (Eiko Engi-neering, model IB-2, Japan). Accelerating voltages of 5 kV was used to observe the morphologies of the gold coated microcapsules. The samples were determined by image processing software (Image J, NIST) and captured by automatic image-capturing software. 

*Overall desirability (OD) function*

To combine the five measured responses in one OD function, individual desirability functions have to be calculated first. Individual desirability function involves transformation of each estimated response variable *Y*_i_ to a desirability value *d*_i_, where 0 ≤ *d*_i_ ≤ 1. The value of *d*_i_ increases as the “desirability” of the corresponding response increases (29). In this work, two methods were used in the calculation of individual desirability functions. 

For response variables that were desired to be maximized, such as yield (w/w, %), EE (%), and AR (%) of microcapsules, *d*_i_ could be calculated as follows (19,20):

**Figure:**
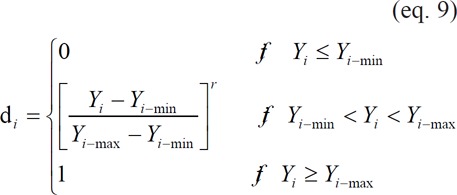


where *Y*_i_ are the actual observed response values of type *i*. *Y*_i-min_ and *Y*_i-max_ are minimum and maximum acceptable values of response *i*, respectively. *r* is a positive constant and is known as weight. For these three response variables, the more general linear-scale desirability function was used, i.e., *r *= 1 (30). Table 1. 

showed the minimum and maximum acceptable values for these three response variables.

For P_1_ and t_85_, the two selected parameters of sustained-release characteristics, we used Harrington’s exponential function (31), which was described as follows: based on the distribution of values, a desirability value (*d*) of 0.4 was assigned to P1 of 8%, and a value of 0.8 was assigned to p1 of 12% in the desirability scale with a maximum of 1.0. Similarly, t_85_ of 5 h was given a desirability value (*d*) of 0.2, and a t_85_ of 12 h was assigned a value of 0.8. Each of these two desirability values (*d*) was transformed to a dimensionless response (*Yʹ*) using the equation: 

*Yʹ *= -[ln(-ln* d*)]                      (eq. 10)

From the two paired values of *Y* and *Yʹ*, the following linear transformation equation was calculated.

*Yʹ *= *b*_0 _+ *b*_1_*Y *                     (eq. 11)

Where *b*_0_ and *b*_1_ are constants, and in this case they were found to be equal to -2.379 and +0.353 for P_1_, and -2.13 and +0.33 for t_85_, respectively. The desirability value of each formulation was calculated from the *Yʹ* value using the exponential equation:

*D *= exp{-[exp[(-*Yʹ*)]}                      (eq. 12)

The individual desirabilities were then combined using the geometric mean. 

OD = (*d*_1_×*d*_2_×…×*d*_k_)^1/^^k^                     (eq. 13)

This single value of OD gives the overall assessment of the desirability of the combined response levels.

*Experimental design and statistical analysis*

The effects of three independent variables, namely pH at complex coacervation (X_1_), gelatin concentration (X_2_, w/w, %), core/wall ratio (X_3_, %), on the OD of multiple response variables, including yield (w/w, %), EE (%), AR (%), and sustained-release profile (P_1_, t_85_) were studied using a three factor central composite design (CCD). Twenty microcapsules samples were established based on the CCD with three independent variables at five levels on each variable. The center point was repeated six times to calculate the reproducibility of the method. Multiple regression analysis was applied for prediction of the linear, quadratic and interaction terms of the independent variables in the RSM. Regression analysis was performed to estimate the response function as polynomial model:

$Y=\beta_{0}+\sum \beta_{i}x_{i}+\sum \beta_{\mathrm{ii}}x_{i}^{2}+\sum \beta_{\mathrm{ij}}x_{i}x_{j}$

(eq. 14)

Where *Y* is response calculated by the model, *β*_0_ is a constant, and *β*_i_, *β*_ii_, and *β*_ij_ are linear, squared and interaction coefficients, respectively (17). Data were modeled by multiple regression analysis. The significant terms in the model were found by analysis of variance for each response. The statistically significant parameters at the 95% significance level were only selected for the model construction. Experimental data were compared with the fitted values predicted by the models in order to verify the adequacy of the regression models. Each experiment was repeated in triplicate.

## Results and Discussion

*Optimization of GCM by RSM*

Table 2. listed the evaluation results of each formulation in terms of the individual responses, along with the calculated OD function values. The results of the applied statistical tests indicated that five response variables measured in this study as well as the OD function showed good fitting to different models. The fitting equations after models were simplified, the corresponding r^2^ values of models, and significance levels of individual parameters were listed in Table 3.

*Yield (%)*

**Figure 1:**
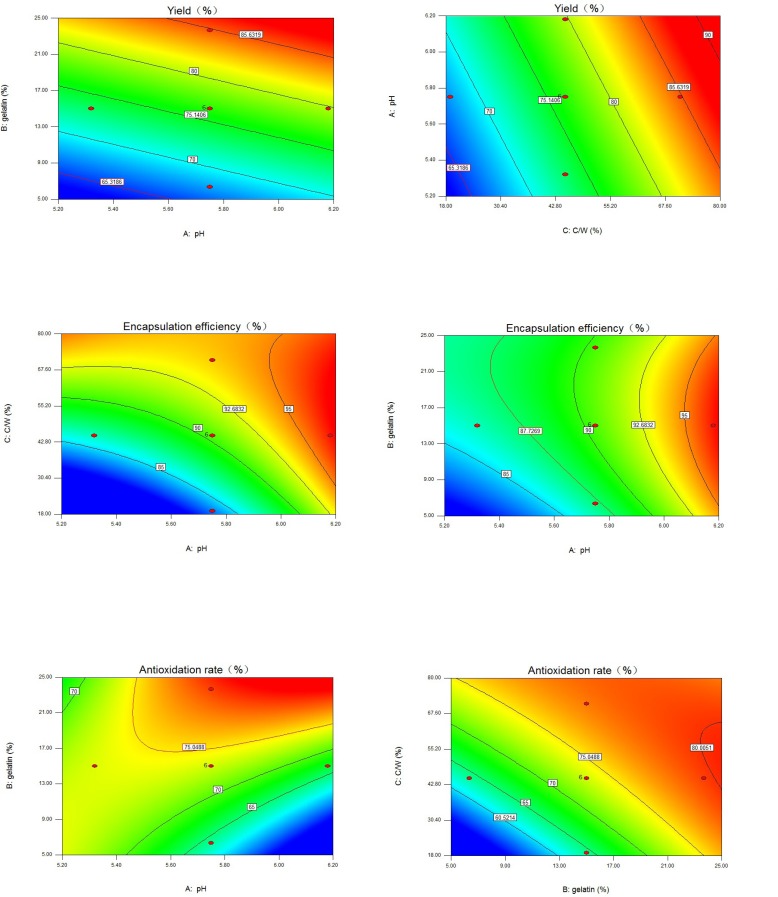
Contour plots of three response variables. a) Contour plot of yield, gelatin concentration *vs.* pH with core/wall ratio = 45%; b) Contour plot of yield, pH *vs.* core/wall ratio with gelatin concentration = 15%; c) Contour plot of encapsulation efficiency, pH* vs.* core/wall ratio with gelatin concentration = 15%; d) Contour plot of encapsulation efficiency, gelatin concentration *vs. *pH with core/wall ratio = 45%; e) Contour plot of antioxidation rate, gelatin concentration *vs.* pH with core/wall ratio = 45%; f) Contour plot of antioxidation rate, core/wall ratio *vs.* gelatin concentration with pH = 5.75. C/W = core/wall ratio.

From the fitting equation (Table 3) and contour plots (Figure. 1a & 1b), yield increased in a linear manner. Gelatin concentration and core/wall ratio had greater influence on yield than pH. The positive coefficient of X_1_ referred to the increase in yield with increasing pH in the experimental range. The results of plot experiments indicated that insufficient reaction between gelatin and chitosan would occur at Ph < 5.30, and pH > 6.30 would bring about the low solubility of chitosan. A narrow range of pH (5.32-6.18) for complex coacervation were selected in this experimental design. In this range, pH had a weaker influence on yield. The yield was also increased by the gelatin concentration increase. Gelatin played an important role in the formation of a stable emulsion because of itself’s emulsification. Studies indicated that beads formed from low concentrations of polymer in solution were expected to be weaker. Random collision and contact of spheres caused blending of weaker particles into each other and resulted in non-uniform shape (32). 

The irregularly-shaped droplets that were formed in the absence or at extremely low concentrations of gelatin could stick to the stirrer, or to one another, forming big lumps, which could reduce the yield, as demonstrated by Chung *et al.* (33). In our previous experiments, the 15-25% concentration of gelatin was enough to precipitate GCM. When the concentration was higher than 25%, it was difficult to gain product due to high viscosity and heavy gelatinization below 25 ºC. Increasing core/wall ratio increased the yield. 

This increase corresponded to the positive coefficients of X_3_. A higher core/wall ratio resulted in less sticky droplets, which reduced the loss caused by droplets aggregation in the post-processing step (34). 

*EE (%)*

EE showed good fitting to the quadratic model (Table 3). The coefficients of X_1_, X_2_, and X_3_ in the fitting equation and the contour plots (Figure. 1c & 1d) indicated that the effects of pH at complex coacervation and core/wall ratio on EE were greater than gelatin concentration. EE increased as pH was increased (positive coefficient of X_1_). This could be attributed to the higher degree of complex coacervation reaction between gelatin and chitosan at higher pH (35, 36). As the core/wall ratio increased, EE significantly increased (positive coefficient of X_3_). A possible explanation for this was that only a fixed amount of AO was probably lost to the external phase during the formation of microcapsules and this loss obviously had a more detrimental effect on EE with relatively lower drug content (37). After reaching a maximum level, EE started to decline slightly as the core/wall ratio was further increased. This was indicated by the negative coefficient of the square term of X_3_ and could be attributed to the practical limitation to the amount of drug that can be incorporated in the microcapsules (38, 39). Within the experimental range, gelatin concentration had the smallest effect on EE; this effect, however, was statistically significant. Increasing gelatin concentration increased EE. The only statistically significant interaction in the EE equation occurred between pH at complex coacervation and core/wall ratio. This interaction was of a negative magnitude.

**Table 1:** Minimum and maximum acceptable levels of the response variables

**Response Variable** **s**	**Minimum Level (%)**	**Maximum Level (%)**
Yield	65	85
EE	75	95
AR	50	80

EE = encapsulation efficiency; AR = antioxidation rate

**Table 2:** Experimental levels of the independent variables and values of the five measured responses and OD function for the prepared GCM formulations

**Run** **Number**	**Independent variables**			**Response variables**					**OD**
	**pH** **(X**_1_ **)**	**g** **elatin** ** conc.** **(X**_2_ **, %)**	**core/wall** **ratio (X**_3_ **,%)**	**Yield** **(** **%** **)**	**EE** **(** **%** **)**	**AR** **(** **%** **)**	**P**_1_ **(** **%)**	**t**_85_ **(h)**	
1	5.75	6.34	45.00	67.06	86.76	62.74	12.70	4.50	0.27
2	5.75	23.66	45.00	87.58	91.13	80.76	8.37	14.50	0.76
3	5.50	20.00	30.00	78.71	83.86	74.15	9.75	11.00	0.50
4	5.75	15.00	19.02	64.00	83.50	64.65	12.87	5.00	0
5	6.18	15.00	45.00	80.61	97.00	66.38	10.66	13.00	0.74
6	5.75	15.00	45.00	78.86	89.01	73.42	9.74	11.50	0.63
7	5.75	15.00	45.00	75.40	90.38	73.00	8.90	12.00	0.60
8	5.75	15.00	45.00	74.97	91.45	73.74	11.03	12.50	0.66
9	6.00	20.00	60.00	87.37	94.69	81.23	6.05	13.50	0.70
10	5.75	15.00	45.00	79.60	90.81	73.21	9.57	11.50	0.66
11	5.32	15.00	45.00	71.14	86.29	74.24	8.99	9.50	0.48
12	6.00	10.00	60.00	78.86	94.06	70.13	9.40	7.50	0.60
13	6.00	10.00	30.00	69.14	90.61	54.61	13.24	5.50	0
14	6.00	20.00	30.00	78.67	90.60	70.88	10.98	12.50	0.66
15	5.50	10.00	30.00	63.94	81.56	67.80	13.31	5.00	0
16	5.50	10.00	60.00	79.57	90.19	75.29	8.41	5.00	0.48
17	5.75	15.00	70.98	83.49	93.99	77.67	6.67	6.50	0.57
18	5.75	15.00	45.00	75.17	91.33	72.39	8.95	12.50	0.62
19	5.50	20.00	60.00	82.67	91.51	73.96	4.85	10.00	0.50
20	5.75	15.00	45.00	74.93	89.81	74.31	10.29	12.00	0.62

GCM = gelatin-chitosan microcapsules; conc. = concentration; EE = encapsulation efficiency; AR = antioxidation rate; P_1_= % of drug released in 1 h; t_85_ = time to 85% drug release; OD = overall desirability.

**Table 3:** The fitting models, equations, and statistical parameters of five measured responses and OD function

**Response Variable** **s**	**Model** **s**	**Equation** **s** a	**r** ^2^	**P** b	**Lack of fit**
Yield (%)	Linear	Yield (%) = +76.59+1.83X_1_+5.10X_2_+5.13X_3_	0.8660	≤0.0001	NSS
EE (%)	Quadratic	EE (%) = +90.47+2.96X_1_+0.84X_2_+3.00X_3_-1.09X_1_X_3_-0.54X_2_^2^-0.60 X_3_^2^	0.9548	≤0.0113	NSS
AR (%)	Quadratic	AR (%) = +73.35-2.00X_1_+4.54X_2_+3.98X_3_+2.79X_1_X_2_+2.32X_1_X_3_-1.61X_2_X_3_-1.04X_1_^2^-0.54X_2_^2^-0.74 X_3_^2^	0.9769	≤0.0031	NSS
P_1_ (%)	Linear	P_1_ (%) = +9.74+0.45X_1_-1.45X_2_-2.09X_3_	0.9153	≤0.0001	NSS
t_85_ (h)	Quadratic	t_85_ (h) = +12.00+1.01X_1_+2.95X_2_+0.33X_3_+0.50 X_1_X_3_-0.27 X_1_^2^-0.85 X_2_^2^-2.10 X_3_^2^	0.9896	≤0.0001	NSS
OD	Quadratic	OD = +0.63+0.067X_1_+0.15X_2_+0.15X_3_+0.03 X_1_X_2_-0.13 X_2_X_3_ -0.047 X_2_^2^-0.12 X_3_^2^	0.9780	≤0.0001	NSS

EE = encapsulation efficiency; AR = antioxidation rate; P_1_= % of drug released in 1 h;

t_85_ = time to 85% drug release; OD = overall desirability; NSS = not statistically significant.

a Where X_1_, X_2_, and X_3_ represent the independent variables: pH at complex coacervation, gelatin concentration (w/w, %), core/wall ratio (%), respectively.

b Significance levels of the individual parameters.

**Figure 2:**
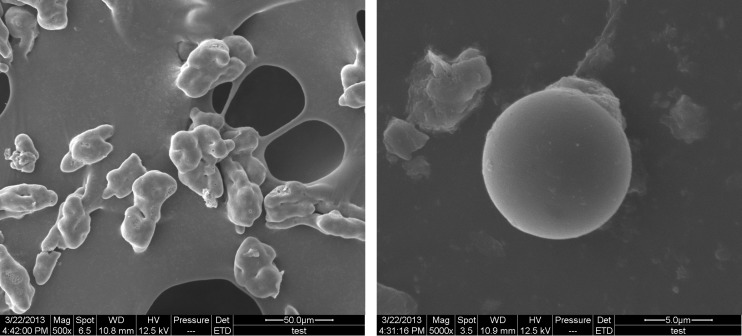
Scanning electron micrograph of gelatin-chitosan microcapsules. a) magnification of 500x; b) magnification of 5000x.

**Figure 3:**
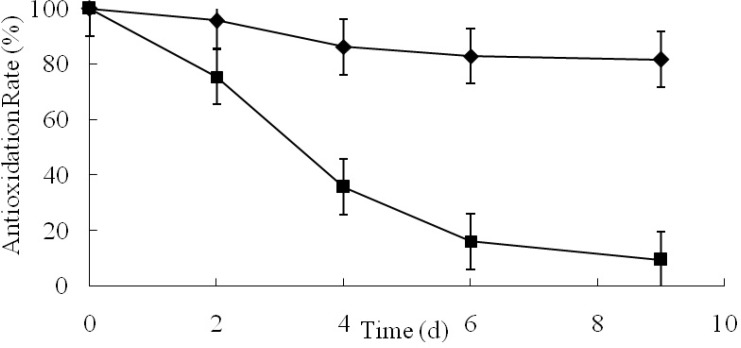
The antioxidative plot of angelica essential oil and microcapsules (■ angelica essential oil ◆ microcapsules).

**Figure 4:**
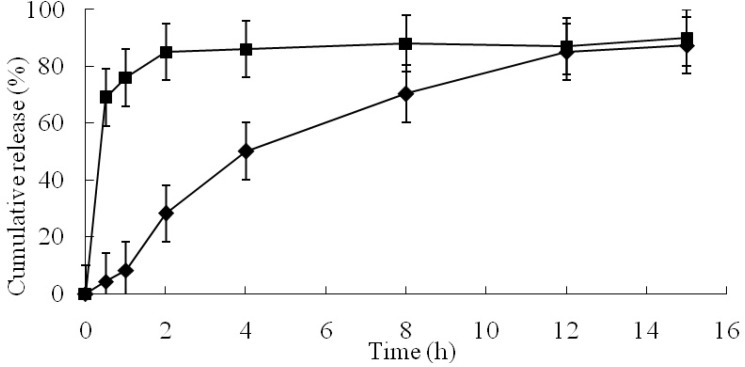
The release profile of angelica essential oil and microcapsules (pH=6.20, gelatin concentration=25.00% (w/w), and core/wall ratio=40.40%). (■ angelica essential oil ◆ microcapsules).

**Figure 5:**
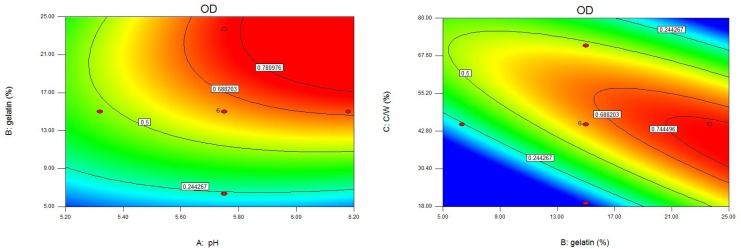
Contour plots of overall desirability as a function of three independent variables. a) gelatin concentration *vs.* pH with core/wall ratio = 45%; b) core/wall ratio *vs.* gelatin concentration with pH = 5.75. C/W = core/wall ratio.

*AR (%)*

The compact degree of surface was an important factor that influenced the AR and sustained-release quality of microcapsules. Studies indicated that the compact surface (Figure. 2) could help to protect core material from oxygen, heat, and so on (40). The AR of microcapsules was significantly influenced by all three variables. This was manifested by square, linear, and interaction terms in the fitting equation (Table 3). AR showed good fitting to the quadratic model. The negative coefficients of X_1_ and X_1_^2^ indicated that AR increased as pH at complex coacervation decreased. This could be attributed to the less compact surface of microcapsules at higher pH (41). Based on the fitting equation, AR was significantly dependent on the gelatin concentration and core/wall ratio. Initially, as the gelatin concentration or core/wall ratio increased, the surface of microcapsules was more compact (42) and AR increased (positive coefficients of X_2_ and X_3_). However, at higher gelatin concentrations or at higher core/wall ratios, AR decreased as gelatin concentration or core/wall ratio increased (negative coefficients of X_2_^2^ and X_3_^2^). This could be related to parallel effects of these factors on EE. 

The AR plots of AO and GCM were shown in Fig. 3. After 9-day acceleration tests, the drug content of GCM was 81.77%, which was much higher than that of AO. The AR of GCM was approximately 8 times higher than one of AO.

*Sustained-release profile (P*_1_ *, t*_85_ *)*

Different release parameters had been used as response variables to describe and optimize the release process. The most frequently used parameters included the percent of drug released at certain time point, the time needed to release a certain percent of the drug, dissolution curve shape factor, dissolution rate constant, and the release order (43). In this study, P_1_ and t_85_ were selected as the response variables to ensure full description of the release profile. P_1_ was used to describe the initial phase of drug release and detect any dose burst effect, while t_85_ could ensure that most of the drug was released in a period of time comparedto the gastrointestinal residence time (44). 

P_1_ was significantly influenced by all three formulation variables in a linear manner. Increasing pH at complex coacervation increased P_1_. As gelatin concentration or core/wall ratio increased, P_1_ significantly decreased. This could be related to an opposite, but parallel effect of these factors on AR, because some studies indicated that the looser surface of microcapsules could result in a marked burst effect and a higher P_1_ value (9,43).

t_85_ was good fitting to quadratic model. It was clear that t_85_ was highly dependent on gelatin concentration. As gelatin concentration increased, the surface of microcapsules was more compact, and t_85_ increased significantly. This corresponded to the higher, positive coefficient of X_2_. This was consisted with the related results of AR and P_1_. However, the negative coefficient of X_3_^2^ indicated a decrease in the t_85_ at extremely high core/wall ratios. 

The *in-vitro* drug release behavior of the newly developed GCM containing AO showed prolonged AO release over 12 h (Figure. 4). The P_1_ of GCM was only 8.29%, but P_1_ of AO reached 76%. This could be attributed to the fact that gelatin and chitosan formed coacervate, which could reduce the release of AO from the inner phase to the outer phase of the GCM (45, 46). Some studies indicated that the particulate hydrogels, based on covalently crosslinked chitosan and gelatin by glutaraldehyde, could be considered good candidates for drug delivery (47, 48). The strong networks formed by the double crosslinking of the two polymers in sustained or controlled release system could prevent a rapid disintegration during initial stage (49).

The determination coefficient (r^2^) values of the zero-order, first-order, and Korsmeyer-Peppas release models were 0.9019, 0.6772, and 0.9486, respectively. The well known Higuchi model (r^2 ^= 0.9767, higher than others) was found to adequately describe the entire release profile (i.e., up to 87% cumulative release) over time. This equation had been used previously to describe release from systems similar to those studied here (21). In addition, Korsmeyer-Peppas model was found to be closer to the best-fit Higuchi model. The values of release exponent (n) determined from *in-vitro* drug release data was 1.08, indicating the drug release from these microcapsules followed by the super case-II transport mechanism controlled by swelling and relaxation of polymeric matrix. This could be attributed due to polymer dissolution and enlargement or relaxation of polymeric chain (50). 

*OD and Prediction*

The use of an OD function allowed prediction of the ranges of independent variables where the preferable formulation(s) could occur. The OD was dependent on all the investigated independent variables. This could be seen in the fitting equation for OD (Table 3), which represented the relations between OD and the independent variables. pH at complex coacervation had a positive impact on the OD (positive coefficient of X_1_). This could be attributed to the increase in yield, EE, P_1_, and t_85_. However, increasing pH decreased AR. The positive effects of gelatin concentration and core/wall ratio on the OD (positive coefficients of X_2_ and X_3_) were the results of their positive effects on yield, EE, AR, and t_85_. However, as gelatin concentration or core/wall ratio increased, P_1_ decreased. Studies had shown that the optimal factor settings for one performance characteristic were not necessarily compatible with those of other performance characteristics. In more general situations, finding compromising conditions on the input variables might be considered that were somewhat favorable to all responses (51, 52). It could be seen from Figure 5. and Table 3. that the highest OD could be achieved at high pH, high gelatin concentration, and intermediate core/wall ratio. The optimized formulation was prepared at pH 6.20, gelatin concentration 25.00%, and core/wall ratio 40.40%. The measured responses (yield 88.51%, EE 94.85%, AR 81.77%, P_1_ 8.29%, and t_85 _15.93 h) of that formulation were very close to the predicted values, and all deviations were less 3%.

Both of two plots (Figure 5) showed that the surface to be relatively flat near the maximum, meaning that small departures from optimality of the variable values, would not appreciably decrease the responses (29).

## Conclusion

An AO-loaded microcapsule formulation with excellent antioxidation and sustained-release properties was optimized using RSM by fitting a second-order model to the response data. The model was found to be satisfactory for describing the relationships between independent variables and individual response variables, as well as the relationships between independent variables and the OD. The use of a desirability function appeared to be a useful approach for handling the problem of multiple responses in this case. The optimization method enabled us to predict the values of response variables and OD within the experimental range with good agreement between the predicted and experimental values. An optimum desirability of GCM was achieved at pH 6.20, gelatin concentration 25.00%, and core/wall ratio 40.40%.
